# Using Multi-Omics Analysis to Explore Diagnostic Tool and Optimize Drug Therapy Selection for Patients with Glioma Based on Cross-Talk Gene Signature

**DOI:** 10.32604/or.2024.046191

**Published:** 2024-11-13

**Authors:** YUSHI YANG, CHUJIAO HU, SHAN LEI, XIN BAO, ZHIRUI ZENG, WENPENG CAO

**Affiliations:** 1 Department of Pathology and Pathophysiology, School of Basic Medicine, Guizhou Medical University, Guiyang, 550025, China; 2 Guizhou Provincial Engineering Technology Research Center for Chemical Drug R&D; State Key Laboratory of Functions and Applications of Medicinal Plants, Guizhou Medical University, Guiyang, 550014, China; 3 Department of Physiology, School of Basic Medicine, Guizhou Medical University, Guiyang, 550025, China; 4 Department of Anatomy, School of Basic Medicine, Guizhou Medical University, Key Laboratory of Human Brain Bank for Functions and Diseases of Department of Education of Guizhou Province, Guizhou Medical University, Guiyang, 550025, China

**Keywords:** Glioma, Cross-talk, Macrophages, Prognosis, Drug therapy selection

## Abstract

**Background:**

The heterogeneity of prognosis and treatment benefits among patients with gliomas is due to tumor microenvironment characteristics. However, biomarkers that reflect microenvironmental characteristics and predict the prognosis of gliomas are limited. Therefore, we aimed to develop a model that can effectively predict prognosis, differentiate microenvironment signatures, and optimize drug selection for patients with glioma.

**Materials and Methods:**

The CIBERSORT algorithm, bulk sequencing analysis, and single-cell RNA (scRNA) analysis were employed to identify significant cross-talk genes between M2 macrophages and cancer cells in glioma tissues. A predictive model was constructed based on cross-talk gene expression, and its effect on prognosis, recurrence prediction, and microenvironment characteristics was validated in multiple cohorts. The effect of the predictive model on drug selection was evaluated using the OncoPredict algorithm and relevant cellular biology experiments.

**Results:**

A high abundance of M2 macrophages in glioma tissues indicates poor prognosis, and cross-talk between macrophages and cancer cells plays a crucial role in shaping the tumor microenvironment. Eight genes involved in the cross-talk between macrophages and cancer cells were identified. Among them, periostin (*POSTN*), chitinase 3 like 1 (*CHI3L1*), serum amyloid A1 (*SAA1*), and matrix metallopeptidase 9 (*MMP9*) were selected to construct a predictive model. The developed model demonstrated significant efficacy in distinguishing patient prognosis, recurrent cases, and characteristics of high inflammation, hypoxia, and immunosuppression. Furthermore, this model can serve as a valuable tool for guiding the use of trametinib.

**Conclusions:**

In summary, this study provides a comprehensive understanding of the interplay between M2 macrophages and cancer cells in glioma; utilizes a cross-talk gene signature to develop a predictive model that can predict the differentiation of patient prognosis, recurrence instances, and microenvironment characteristics; and aids in optimizing the application of trametinib in glioma patients.

## Introduction

Glioma, a primary cranial malignant tumor originating from glial cells, exhibits a global incidence rate of 4.6 to 5.7 per 100,000 individuals, with a notable increase in recent years [1]. Gliomas are observed across all age groups, with a higher incidence in adults, particularly among males [2]. In 2016, the World Health Organization introduced a histological classification of gliomas, categorizing them into grades I–IV. Initially, researchers hypothesized that glioma grade correlated with increased malignancy and poorer patient prognosis. However, advances in the understanding of glioma molecular biology have revealed that the tumor grade of patients with glioma is not an independent prognostic factor. Individuals with the same pathological grades may exhibit varying survival rates and prognoses because of their dissimilar genetic backgrounds [3]. Through extensive examination of the molecular gene profile of individuals with glioma, researchers have discovered that certain molecular markers, such as isocitrate dehydrogenase 1 *(IDH1*) mutations and O-6-methylguanine-DNA methyltransferase (*MGMT*) promoter methylation, possess dual functionality as both prognostic indicators and criteria for assessing drug resistance [4,5]. Consequently, the identification of a novel molecular biomarker for glioma is of utmost significance as it has the potential to profoundly influence therapeutic strategies and prognostic results.

Previous studies have suggested that various components of the tumor microenvironment (TME), such as hypoxia, acidosis, inflammation, and immune status, interact with one another and play a role in the growth of glioma as well as in influencing the response to treatment [6,7]. For instance, Guo et al. revealed that a hypoxic microenvironment promotes the infiltration of tumor-associated macrophages and induces M2 polarization within glioma tissues, thereby facilitating glioma proliferation [8]. Sinha et al. demonstrated that the activation of inflammatory pathways, specifically the TNFα-related pathway, in glioma cells plays a role in regulating radiosensitivity by increasing the levels of reactive oxygen species [9]. Wang et al. proposed that the NF-κB inflammatory pathway enhances the transcription of PD-L1, leading to immune evasion [10]. Numerous genes implicated in the alteration of the TME have been shown to influence patient prognosis. For instance, sorting nexin 20 has been linked to immune cell infiltration and serves as a prognostic biomarker for gliomas [11]. Additionally, hexokinase 3 has the ability to recruit immune cells to the TME, thereby facilitating glioma progression [12]. Therefore, the identification of biomarkers associated with TME characteristics could potentially aid in prognostic evaluation and selection of appropriate therapeutic drugs.

Immune cells are integral components of the TME and are regulated by diverse microenvironmental factors such as hypoxia and inflammation. Their involvement in the progression and drug resistance of glioma cells is well established [13,14]. Consequently, this study prioritized immune cells and sought to establish biomarkers for glioma diagnosis and treatment selection based on the interplay between immune and cancer cells. Our study revealed a significant association between M2 macrophages and an unfavorable prognosis in glioma. Additionally, we found that the interaction between macrophages and cancer cells played a crucial role in shaping the glioma tissue microenvironment. Four genes involved in cross-talk, periostin (*POSTN*), chitinase 3 like 1 (*CHI3L1*), serum amyloid A1 (*SAA1*), and matrix metallopeptidase 9 (*MMP9*), were used to construct a predictive model for prognosis. Moreover, this model has the potential to differentiate between high inflammation and hypoxia–immunosuppressive microenvironment signatures and aid in optimizing drug selection for glioma treatment.

## Materials and Methods

### Acquisition of TCGA and CGGA cohorts

The gene expression matrix of glioma tissues was obtained from The Cancer Genome Atlas (TCGA; https://portal.gdc.cancer.gov/) and Chinese Glioma Genome Atlas (CGGA; http://www.cgga.org.cn/). The TCGA cohort was used to investigate primary biomarkers and develop a primary risk model, therefore it was designated as the training cohort. The CGGA cohort was employed to conduct a secondary validation of the effects of the risk model and was designated as the testing cohort. The TCGA and CGGA datasets, containing 662 and 313 glioma patients, respectively, were included in this study. Only patients with complete clinical information were included. Prior to analysis, probe names were converted to gene symbols, and batch normalization was performed on the gene expression matrices. Genes with more than 50% missing values were excluded from the analysis.

### Calculation of the levels and effects of immune cells on glioma patient survival

CIBERSORT, an R package developed by Chen et al. [15], uses a deconvolution algorithm to measure 22 cell fractions from gene expression matrices of tissues. The gene expression profile was represented as a level matrix of 22 immune cells. To determine changes in cell types, one-way analysis of variance combined with Tukey’s test was employed for surviving patients, patients who had died with overall survival (OS) time ≥14 months (above median survival), and patients who had died with OS time <14 months (lower than median survival) [16]. The relationship between the 22 immune cell types and survival of patients with glioma was determined using log-rank Kaplan–Meier (KM) analysis. Statistical significance was set at *p* < 0.05.

### Differentially expressed genes (DEGs) analysis

The Limma package was used to assess differentially expressed genes (DEGs) between glioma tissues characterized by high and low M2 macrophage levels in the R software environment. DEGs were identified using a cut-off criterion of log2 fold change (FC) ≥ 1 and adjusted *p* < 0.05. The volcano plot displayed alterations in gene expression for all genes, whereas the heatmap plot specifically highlighted DEGs.

### Single cell RNA (scRNA) sequencing analysis

The scRNA sequencing cohort GSE103224 was obtained from the Gene Expression Omnibus (GEO) dataset (https://www.ncbi.nlm.nih.gov/gds) and the data were analyzed using the Seurat package. Initially, high-quality cells meeting the criteria of 200 < nFeature < 6000 and mitochondria (MT) content < 3 were selected for further analyses. A subset comprising 2000 genes exhibiting the highest variability was chosen for analysis. Principal component analysis (PCA) was subsequently employed, leading to the identification of 15 distinct PCA signatures. The selection of the PCA number was contingent upon a notable disparity in standardized variance between the cutoff thresholds and the adjacent thresholds. These signatures were subsequently used to conduct cell clustering with a resolution parameter of 0.5. Cell annotation was performed using CellMarker 2.0 (http://bio-bigdata.hrbmu.edu.cn/CellMarker/) by employing the top ten signature genes associated with each cell cluster. Next, the cell clusters were consolidated, and genes with high abundance in each cell type were chosen using a criterion of expression in 25% of the cell type and a 1.25-fold change relative to the expression levels in other cells. Cell communication was examined using the scConnect software package.

### Construction and verification of a predictive model

The prognostic value of genes in patients with glioma in TCGA was analyzed using univariate Cox regression analysis, and the cut-off value was set at *p* < 0.05 to determine significant. Significant genes were analyzed to remove collinear genes using LASSO analysis. Based on the Akaike information criterion (set as 2426.12), an optimal prognostic risk index was constructed to divide patients with glioma into high- and low-risk groups after calculating the coefficient using multivariate Cox regression analysis. KM analysis was used to analyze the survival differences between high- and low-risk groups in both TCGA and CGGA cohorts, and *p* < 0.05 was set as the threshold. The prognostic effectiveness of the predictive model for 3-year survival was analyzed by receiver operator characteristic curve (ROC), while area under the curve (AUC) ≥ 0.70 was set as significant.

### Nomogram construction

Data from patients with glioma in TCGA and CGGA were merged. Information on age, sex, grade, 1p/19q co-deletion, *MGMT* methylation, *IDH1* mutation, and risk score of each patient with glioma was used to conduct multivariate Cox regression analysis, which was then integrated and visualized as a nomogram. The checkpoints of the nomogram were set at 1, 3 and 5-years to confirm its predictive efficiency.

### Tissue collection and immumohistochemical staining (IHC)

Sixty glioma tissue samples were procured from the Affiliated Hospital of Guizhou Medical University (Guiyang, China) following approval from the Human Ethics Committee of Guizhou Medical University (approval number: 2023-272). Written informed consent was obtained from all the participants, who did not receive chemotherapy or radiotherapy before tissue collection during surgery. After the surgical procedure, all patients received standard temozolomide (TMZ) regimens (150–200 mg/m^2^ given once daily on days 1–5 every 4 weeks; combined with radiotherapy for 4 weeks). Of the total cohort, 48 individuals experienced recurrence within one year of TMZ treatment, while 18 individuals did not exhibit recurrence. The expression levels of POSTN, CHI3L1, SAA1, MMP9, carbonic anhydrase 9 (CA9), interleukin 2 (IL-2), and interleukin 6 (IL-6) in glioma samples collected during surgery were reviewed using IHC. IHC was performed as described in our previous study [17], using the following antibodies: anti-POSTN (1:4000; Cat no. 66491-1-Ig, Proteintech, Wuhan, China), anti-CHI3L1 (1:200; Cat no. 12036-1-AP, Proteintech), anti-SAA1 (1:200; Cat no. ab190802, Abcam, USA), anti-MMP9 (1:100; Cat no. 10375-2-AP, Proteintech), CA9 (1:50; Cat no. 11071-1-AP, Proteintech), IL-2 (1:100; Cat no. 26156-1-AP, Proteintech) and IL-6 (1:200; Cat no. 21865-1-AP, Proteintech) antibodies.

### ESTIMATE and tumor immune dysfunction and exclusion (TIDE) analysis

The landscape of glioma tissues, including stromal, immune, and estimate scores in gliomas, was analyzed using R software with the ESTIMATE algorithm. The differences in stromal score, immune score, and ESTIMATE score between the high- and low-risk groups were determined by the unpaired *t*-test with a cut-off of *p* < 0.05. The online TIDE database (http://tide.dfci.harvard.edu/) was used to measure the dysfunction score, exclusion score, TIDE score, and response rate of the gliomas. The differences in dysfunction score, exclusion score, and TIDE score in the high- and low-risk groups were analyzed by unpaired *t*-test with a cut-off of *p* < 0.05, while differences in immune checkpoint blockade (ICB) response rate were analyzed by the chi-square test with a cut-off of *p* < 0.05.

### Gene set variation analysis (GSVA)

A gene set of 50 hallmark pathways of cancers were accessed from Gene Set Enrichment Analysis (GSEA) database (http://www.gsea-msigdb.org/gsea/index.jsp) via applying an index word as “h.all.v7.4. symbols.gmt”. The GSVA package (version 1.40.1) in the R environment was used to calculate the signature of the pathways in each glioma sample according to the rank ordering of genes.

### Drug score calculation

OncoPredict is an R algorithm developed by Maeser et al. [18] that predicts *in vivo* anti-tumor drug responses. The sensitivity of gliomas to 198 drugs was calculated using the OncoPredict script by matching the gene expression matrix of each glioma sample to the cytotoxic effects of drugs in cancer cells recorded in the Genomics of Drug Sensitivity in Cancer and the gene expression information of cancer lines recorded in the Broad Institute Cancer Cell Line Encyclopedia. A high drug score indicates a low sensitivity of patients with glioma to drugs.

### Cell culture and qRT-PCR experiment

Normal human astrocytes (NHA) and glioma cell lines (U87, U251, U118, A172, T98G, SF295, LN229, and SF126) were procured from ATCC (Manassas, VA, USA) and cultured in RMPI-1640 medium supplemented with 10% fetal bovine serum (FBS). All cells were cultured in a controlled environment at a temperature of 37°C with 5% CO_2_. All cells were appraised by STR and performed mycoplasma testing to eliminate latent contamination. Briefly, the cells were lysed using TRIZOL reagent (Yeasen, Shanghai, China) to extract total RNA. The Hifair^®^ III 1st Strand cDNA Synthesis SuperMix (Yeasen, Shanghai, China) and TB green (Yeasen, Shanghai, China) were utilized for reverse transcription and PCR amplification, respectively. Relative expression of the target genes was normalized to that of ACTB. The primers employed in this study were as follows: POSTN-forward, 5′-CTCATAGTCGTATCAGGGGTCG-3′; POSTN-reverse, 5′-ACACAGTCGTTTTCTGTCCAC′; CHI3L1-forward, 5′-GTGAAGGCGTCTCAAACAGG-3′; CHI3L1-reverse, 5′-GAAGCGGTCAAGGG-CATCT-3′; SAA1-forward, 5′-TGCCTGGGCTGCAGAAGTGA-3′; SAA1-reverse, 5′-TGATCAGCCAGCGAGTCCTC-3′; MMP9-forward, 5′-TGTACCGC-TATGGTTACACTCG-3′; MMP9-reverse, 5′-GGCAGGGACAGTTGCTTCT-3′; ACTB-forward, 5′-CATGTACGTTGCTATCCAGGC-3′; ACTB-reverse, 5′-CATGTACGTTGCTATCCAGGC-3′.

### Cell proliferation detection

Glioma cell proliferation was determined using the CCK-8, EDU and 3D sphere formation assays. For the CCK-8 assay, total of 5 × 10^3^ cells was suspended in 100 μL medium and placed in a 96-well plate. After culturing for 48 h with or without 10 nM trametinib, 10 μL of CCK-8 reagent was added to each well and incubated for 2 h. The absorbance of each well was measured at 450 nm wavelength. EDU experiments were performed according to the manufacturer’s instructions (Click-It EdU Imaging Kits) provided by the manufacturer (Invitrogen, USA). In the 3D sphere formation experiment, a total of 500 glioma cells were suspended in 100 μL culture medium and placed in ultra-low adsorption petri dishes (Invitrogen, USA). After a period of 20 days with or without 10 nM trametinib, the condition of the spheres was documented.

### Statistical analysis

All statistical analyses were performed using SPSS software (version 20.0). An unpaired *t*-test was used to determine differences between groups. Statistical significance was set at *p* < 0.05.

## Results

### IL-8, POSTN, CHI3L1, SAA1, PLA2G2A, TREM1, IBSP and MMP9 expression are elevated in glioma tissues with high level M2 macrophages

**Figure 1:**
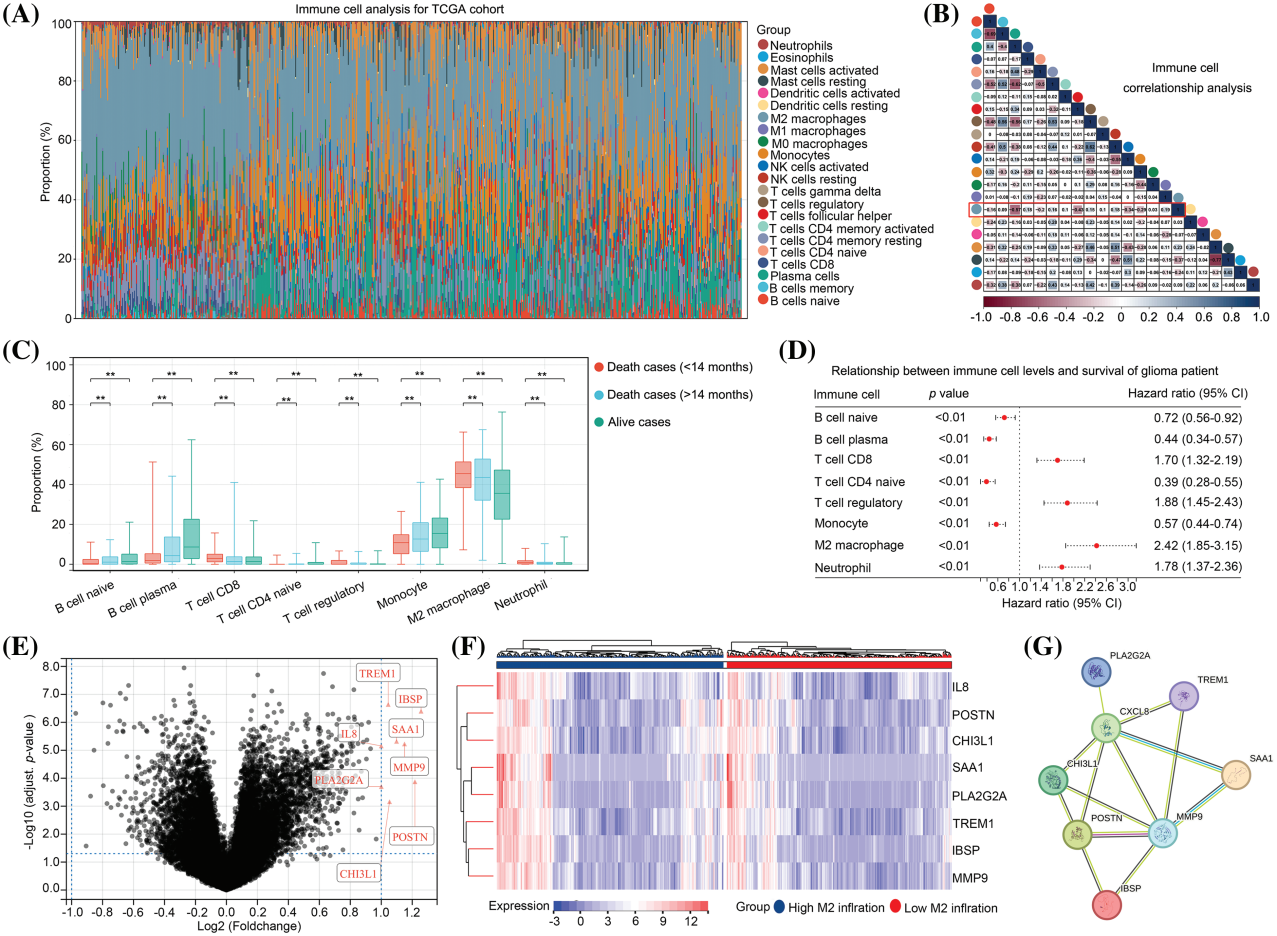
Expression of *IL-8*, *POSTN*, *CHI3L1*, *SAA1*, *PLA2G2A*, *TREM1*, *IBSP* and *MMP9* is elevated in glioma tissues with high level M2 macrophages. (A) The gene expression matrix of glioma tissues in TCGA was transformed into expression levels of 22 immune cells, through CIBERSORT. (B) Co-expression relationship between immune cells. (C) Expression in naïve B cells, plasma B cells, CD4 naïve T cells, monocyte cells, CD8 T cells, regulatory T cells, M2 macrophages and neutrophil in surviving patients, deceased patients with OS ≥ 14 months and deceased patients with OS < 14 months. (D) The effects of the naïve B cells, plasma B cells, CD4 naïve T cells, monocyte cells, CD8 T cells, regulatory T cells, M2 macrophages and neutrophil on the survival rate of patients with glioma in TCGA were analyzed via Kaplan–Meier survival analysis. (E) Volcano plot exhibiting the DEGs in glioma tissues between groups with a high and low infiltration of M2 macrophages. (F) Heatmap plot exhibiting the upregulated genes in glioma tissues between group with a high and low infiltration of M2 macrophages. (G) The protein-protein interaction network indicated the relationships between up-regulated genes. **p < 0.01.

Initially, we focused on the immune cell signatures of gliomas. Consequently, the gene expression profile of glioma tissues from TCGA database was transformed into a matrix consisting of 22 immune cell types using the CIBERSORT algorithm (Fig. 1A). Notably, our analysis revealed that M2 macrophages exhibited the highest abundance among the 22 immune cell types within glioma tissues (Fig. 1A). Furthermore, we observed a negative correlation between M2 macrophages and plasma cells, follicular helper T cells, and activated NK cells in glioma tissues (Fig. 1B). Furthermore, our findings indicated that there was a higher presence of naïve B cells, plasma B cells, CD4 naïve T cells, and monocytes in both surviving and deceased patients with an overall survival (OS) of 14 months or more compared to deceased patients with an OS of less than 14 months. Conversely, the numbers of CD8 T cells, regulatory T cells, M2 macrophages, and neutrophils decreased (Fig. 1C). We subsequently employed KM analysis to examine the impact of these eight cell types on the survival of patients with gliomas. The results revealed that the levels of naïve B cells, plasma B cells, CD4 naïve T cells, and monocytes were inversely associated with unfavorable prognosis in patients with glioma, whereas the levels of CD8 T cells, regulatory T cells, M2 macrophages, and neutrophils were positively correlated with poor prognosis (Fig. 1D). Notably, M2 macrophages exhibited the highest hazard ratio (HR = 2.42, 95% CI = 1.85–3.15) for survival in patients with glioma (Fig. 1D). Subsequently, our investigation centered on the examination of gene expression disparities between glioma tissues exhibiting high and low levels of M2 macrophages (Suppl. Table 1). A total of eight up-regulated genes, namely *TREM1*, *IBSP*, *SAA1*, *IL-8*, *MMP9*, *PLA2G2A*, *POSTN*, and *CHI3L1*, were found in the glioma tissues with elevated M2 macrophages, employing a threshold of |LogFC| ≥ 1 and an adjusted *p* value < 0.05 (Figs. 1E and 1F). Interestingly, expression levels of all eight genes were significantly correlated with one another (Fig. 1G). Therefore, these eight genes were set as M2-related genes and used for further analysis.

### IL-8, POSTN, CHI3L1, SAA1, PLA2G2A, TREM1, IBSP and MMP9 are cross-talk genes for macrophages and cancer cells

**Figure 2:**
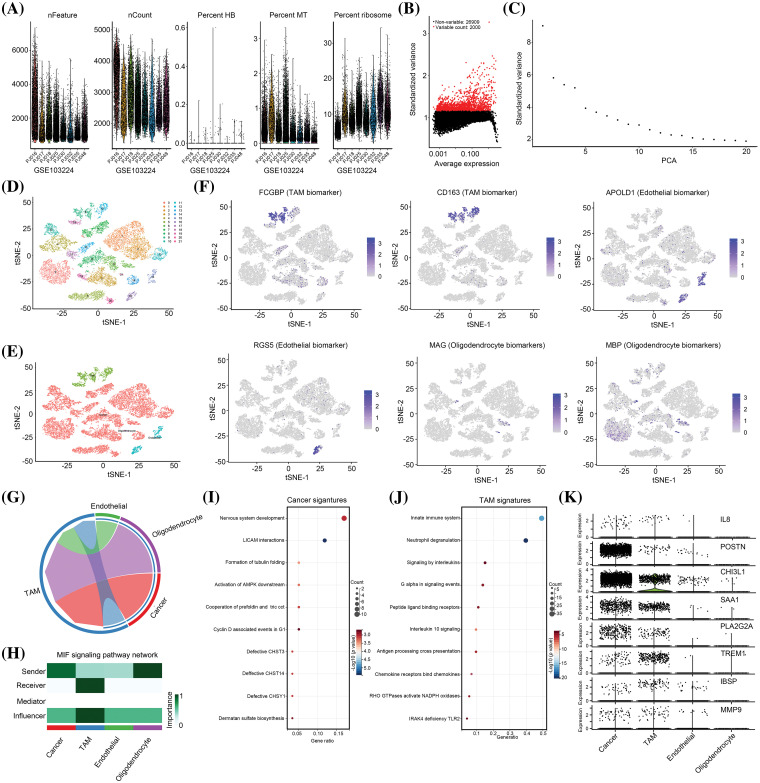
*IL-8*, *POSTN*, *CHI3L1*, *SAA1*, *PLA2G2A*, *TREM1*, *IBSP* and *MMP9* are cross-talk genes between macrophages and cancer cells. (A) Feature, count, hemoglobin (HB), mitochondria (MT), and ribosome level in each cell from GSE103224 cohort. (B) The 2000 genes with the highest frequency of variation from GSE103224 were identified. (C) PCA signatures were used to perform cell cluster analysis. (D) A total of 22 cell clusters were identified in GSE103224. (E) A total of four cell types including TAM, endothelial cells, cancer cells and oligodendrocytes were identified after cell annotation. (F) tSNE plots show the distribution of MAG (oligodendrocyte biomarker), MBP (oligodendrocyte biomarker), APOLD1 (endothelial biomarker), RGS5 (endothelial biomarker), FCGBP (TAM signature) and CD163 (TAM signature). (G and H) Cell communication analysis was performed to analyze the importance and role of TAM, endothelial cells, cancer cells and oligodendrocytes in the TME. (I) Reactome pathway enrichment analysis for top100 signature of cancer. (J) Reactome pathway enrichment analysis for top100 signature of TAM. (K) Cell-type location for expression of *IL-8*, *POSTN*, *CHI3L1*, *SAA1*, *PLA2G2A*, *TREM1*, *IBSP* and *MMP9*.

Although IL-8, POSTN, CHI3L1, SAA1, PLA2G2A, TREM1, IBSP, and MMP9 levels were elevated in glioma tissues with high levels of M2 macrophages, their cell location remained unclear. Therefore, to analyze cell location, a single-cell RNA sequencing cohort (GSE103224) was utilized. Initially, the nFeature, nCount, hemoglobin (HB) percentage, mitochondrial (MT) content, and ribosome content were analyzed in each cell (Fig. 2A). Subsequently, only high-quality cells that met the criteria of 200 < nFeature < 6000 and MT content < 3 were selected for further analysis (Fig. 2A). After selecting the 2000 genes with the highest gene variance (Fig. 2B), the PCA signature of these 2000 genes was calculated. The results indicated that 15 PCA signatures exhibited high variance and were suitable for cell clustering (Fig. 2C). Subsequently, these 15 PCA signatures were used to perform cell clustering, which led to the identification of 22 cell clusters (Fig. 2D). Based on the gene signature, these 22 cell clusters were annotated as four distinct cell types: tumor-associated macrophages (TAM), cancer cells, endothelial cells, and oligodendrocytes (Fig. 2E). To validate the precision of the annotation, we examined the distribution of cell-specific biomarkers, which revealed that all biomarkers were exclusively localized within their respective cell types (Fig. 2F). Subsequently, we analyzed the intercellular communication within these cells, identifying TAM as the most significant contributor (Fig. 2G). Notably, a substantial portion of TAM-mediated communication involved interactions between TAM and both cancer cells and oligodendrocytes (Fig. 2G). Specifically, in the cancer cell cross-talk, we observed that cancer cells assumed a pivotal role as senders, whereas TAM functioned as crucial receivers and influencers (Fig. 2H). We extracted the top 100 cancer cell and TAM signatures (Suppl. Table 2). We found cancer cell signatures were enriched in “nervous system development”, “LICAM interactions”, “formation of tubulin folding”, “activation of AMPK downstream”, “cooperation of prefoldin and tric cct”, “Cyclin D associated events in G1”, “defective CHST3, CHST14 and CHSY1” and “dermatan sulfate bio-synthesis” pathways (Fig. 2I). The TAM signatures were enriched in pathways “innate immune system,” “neutrophil degranulation,” “signaling in interleukins,” “G alpha in signaling events,” “peptide ligand binding receptors,” “IL-10 signaling,” “antigen processing cross presentation,” “chemokine receptors binding chemokines,” “RHO GTPases activate NAPDH oxidases” and “IRAK4 deficiency TLR2” (Fig. 2J). Analysis of the distribution of IL-8, POSTN, CHI3L1, SAA1, PLA2G2A, TREM1, IBSP, and MMP9 revealed that POSTN, CHI3L1, SAA1, and PLA2G2A were primarily expressed in cancer cells, with secondary expression observed in TAM (Fig. 2K). Conversely, TREM1 and IBSP exhibited primary expression in TAM and secondary expression in cancer cells (Fig. 2K).

Furthermore, the overall abundances of IL-8 and MMP-9 were low, with a higher prevalence of expression observed in both TAM and tumor cells (Fig. 2K). Hence, we hypothesized that these eight genes may serve as pivotal mediators in the interplay between TAM and cancer cells. Consequently, all these genes were designated as cross-talk genes between TAM and cancer cells, and were subsequently subjected to further analysis.

### Predictive model constructed with POSTN, CHI3L1, SAA1 and MMP9 has prognostic value for patients with glioma

**Figure 3:**
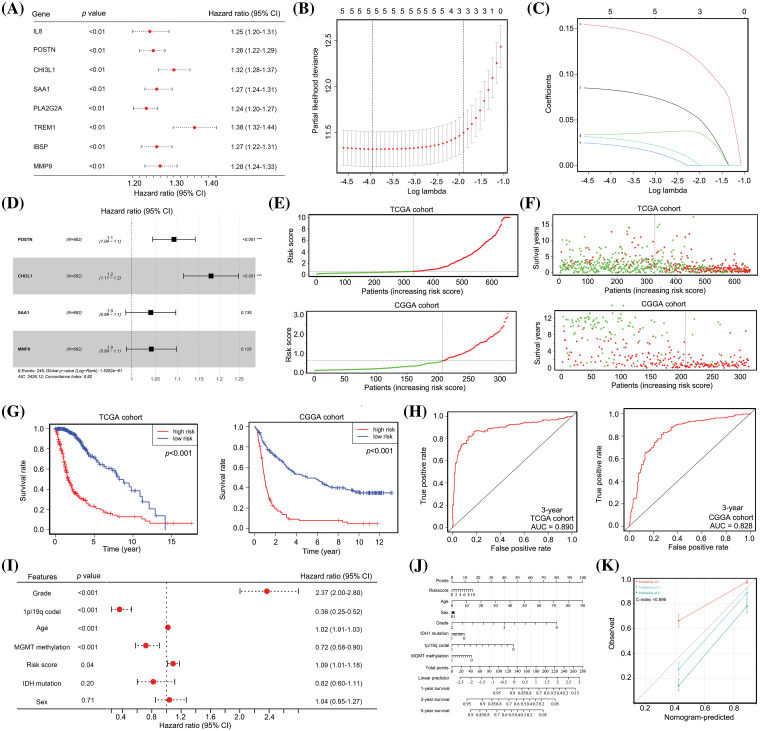
The predictive model, constructed from *POSTN*, *CHI3L1*, *SAA1* and *MMP9* expression, demonstrates significant prognostic value for patients with glioma. (A) Univariate Cox regression analysis for the eight cross-talk genes between TAM and cancer cells. (B and C) LASSO analysis was used to remove homologous cross-talk genes between TAM and cancer cells. (D) Multivariate Cox regression analysis were used to analyze significant cross-talk genes between TAM and cancer cells and to construct a predictive model. (E) Patients with glioma in TCGA and CGGA cohorts were placed into high and low risk groups according to the predictive model. (F) TCGA data revealed that the high-risk group had 190 deaths (red dots) and 141 survivors (green dots), whereas the low-risk group had 55 deaths and 276 survivors. For the CGGA cohort, the high-risk group had 95 deaths and 11 survivors, while the low-risk group had 123 deaths and 84 survivors. (G) Patients with glioma in TCGA and CGGA cohorts in the high-risk group exhibited a lower survival rate. (H) The diagnostic values of the predictive model for survival for patients with glioma in TCGA and CGGA cohorts were 0.890 and 0.828. (I) Multivariate Cox regression analysis for age, sex, tumor grade, *IDH1* status, *MGMT* promoter status, 1p/19q co-deletion and risk score in patients with glioma. (J and K) A nomogram was constructed using the age, sex, tumor grade, *IDH1* status, 1p/19q co-deletion, *MGMT* promoter status, and risk score.

Given the significant impact of cross-talk between TAM and cancer cells on the TME of glioma tissues, our study aimed to develop a predictive model for the prognosis of patients with glioma. To achieve this, we employed univariate Cox regression analysis, which revealed a negative correlation between the expression of all eight cross-talk genes and the survival of patients with glioma (Fig. 3A). Subsequently, we employed LASSO analysis to eliminate collinearity among the genes, resulting in the selection of five genes (*POSTN*, *CHI3L1*, *SAA1*, *TREM1*, and *MMP9*) for further analyses (Figs. 3B and 3C). Multivariate Cox regression analysis indicated that *POSTN* and *CHI3L1* were positively associated with poor prognosis of gliomas (Fig. 3D). Subsequently, a predictive model (risk score = 0.09 × POSTN + 0.16 × CHI3L1 + 0.04 × SAA1 + 0.04 × MMP9) was developed based on their respective coefficient values.

The prognostic value of this predictive model was assessed in both the train cohort (TCGA) and the test cohort (CGGA). Based on the assessment of *POSTN*, *CHI3L1*, *SAA1*, and *MMP9* expression levels in patients with glioma, the risk score was computed for each patient, leading to the classification of patients into high-risk and low-risk categories; the cut-off to determine high and low risk was set as the median risk score obtained from TCGA cohort (Fig. 3E). In both the TCGA and CGGA cohorts, patients with glioma classified as high-risk demonstrated a higher incidence of mortality (Fig. 3F). Furthermore, patients with high-risk gliomas exhibited a shorter survival duration than those classified as low-risk (Fig. 3G). ROC analysis revealed that the model exhibited diagnostic values of 0.890 and 0.828 for predicting 3-year survival in the TCGA and CCGA cohorts, respectively (Fig. 3H).

Our analysis revealed that the high-risk group from TCGA and CGGA cohorts consisted of 14.7%, 36.2%, and 49.1% of patients in stages II, III, and IV, respectively, whereas the low-risk group comprised 53.1%, 36.6%, and 10.3% of patients in stages II, III, and IV, respectively (Suppl. Table 3). Subsequently, multivariate Cox regression analysis was performed to assess the potential independence of the predictive model as a factor for survival of patients with glioma. This analysis incorporated information regarding tumor grade, age, sex, 1p/19q co-deletion status, *IDH1* mutation status, *MGMT* methylation status, and risk score. Evidence suggested that there is a positive relationship between 1q/19p co-deletion and *MGMT* methylation status and OS, while tumor grade, age, and risk score were negatively correlated with OS (Fig. 3I). The findings also indicate that these factors can independently predict the prognosis of patients with gliomas. To aid healthcare professionals in making more informed clinical judgments, a nomogram was constructed using information on risk score, grade, 1q/19p co-deletion, age, *MGMT* methylation, *IDH1* mutation, and sex of patients with glioma (Fig. 3J). This nomogram demonstrated a diagnostic value with a C-index of 0.896 (Fig. 3K). Taken together, our evidence indicates that the predictive model constructed using *POSTN, CHI3L1*, *SAA1* and *MMP9* expression had significant prognostic value for patients with glioma.

### The predictive model has significant diagnostic value for predicting recurrence after TM) treatment

**Figure 4:**
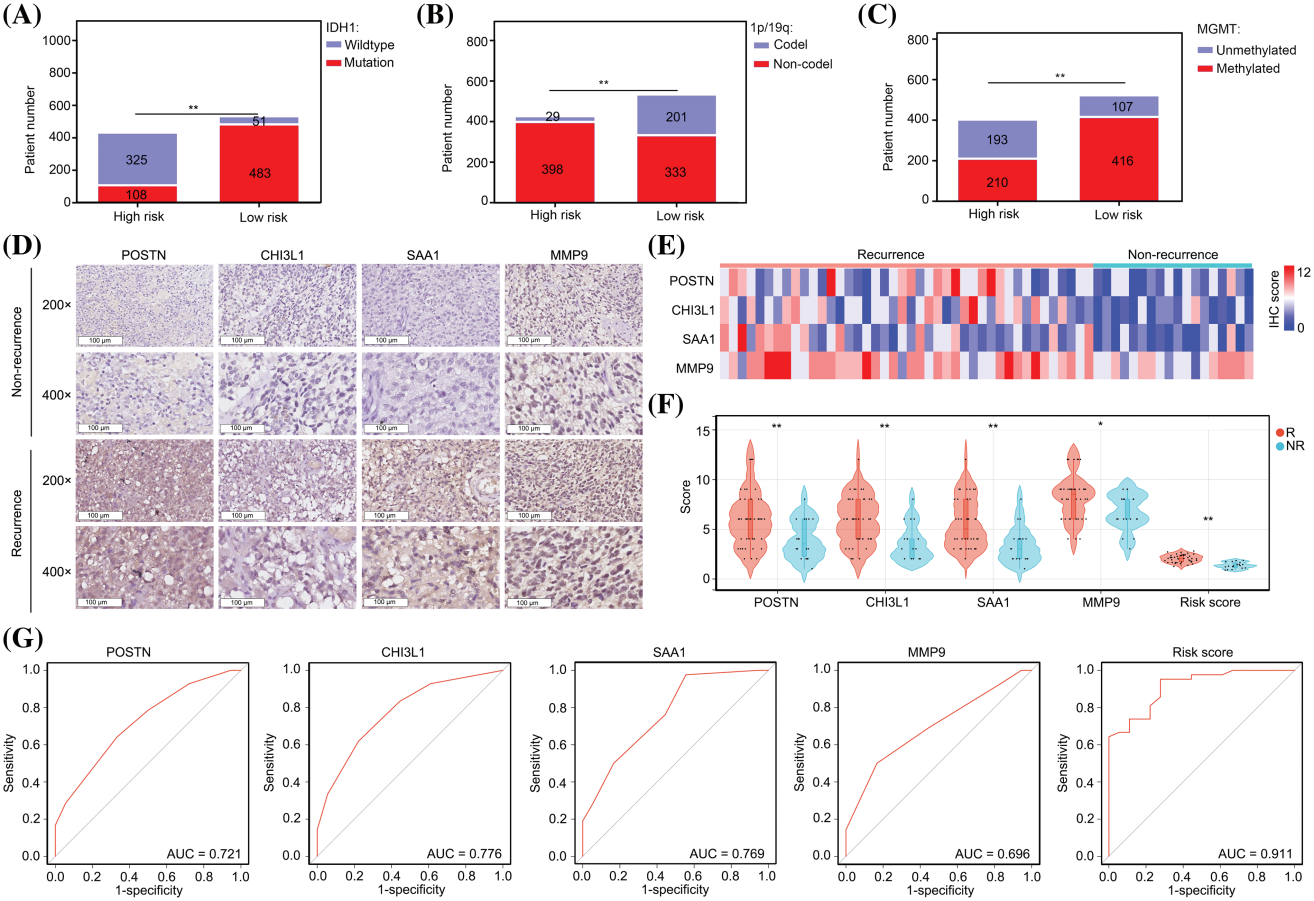
The predictive model has significant diagnostic value for predicting recurrence after TMZ treatment. (A) Patient numbers with *IDH1* mutations in low-risk and high-risk glioma groups. (B) Patient numbers with 1p/19q co-deletion in low-risk and high-risk glioma groups. (C) Patient numbers with *MGMT* promoter methylation in low-risk and high-risk glioma groups. (D–F) High levels of POSTN, CHI3L1, SAA1 and MMP9 expression were found in glioma tissues from patients who experienced recurrence within one year after TMZ treatment. R: recurrence; NR; non-recurrence. (G) Diagnostic value of single genes and risk score to predict recurrence within one year after TMZ treatment. *p < 0.05; **p < 0.01.

Patients with glioma exhibit a diverse array of genetic mutations, including *IDH1* mutation, *MGMT* promoter methylation, and 1p/19q co-deletion [19]. These genetic mutations have been associated with various clinical characteristics, such as recurrence and drug resistance, in patients with glioma. Notably, our study revealed a significant correlation between *IDH1* wildtype genotype (Fig. 4A), 1p/19p co-deletion (Fig. 4B), and *MGMT* promoter methylation status (Fig. 4C) in patients with glioma and a high-risk score. These findings provide compelling evidence that our model can accurately predict the genetic mutation status of patients with gliomas.

As the presence of the 1p/19q co-deletion and *MGMT* promoter methylation are indicative of the sensitivity of patients to TMZ [20], we investigated the value of the model in predicting TMZ sensitivity in patients with glioma. By IHC, we observed elevated expression levels of POSTN, CHI3L1, SAA1, and MMP9 in tissues obtained from patients who experienced recurrence within one year of TMZ treatment (Figs. 4D–4F)

Notably, the predictive values of POSTN, CHI3L1, SAA1, and MMP9 for recurrence after TMZ treatment were 0.721, 0.776, 0.769, and 0.696, respectively (Fig. 4G). We calculated the risk value by incorporating the IHC scores of individual genes into the prediction model formula. Notably, we observed elevated risk scores in glioma tissues obtained from patients who experienced recurrence within one year of TMZ treatment (Fig. 4F). Furthermore, the diagnostic value of the risk score was 0.911, which surpassed that of each signature gene (Fig. 4G). These findings strongly suggest that our model has substantial diagnostic potential for predicting cases of recurrence following TMZ treatment.

### The predictive model has significant diagnostic value for distinguishing high hypoxia and high inflammation microenvironments in glioma tissues

**Figure 5:**
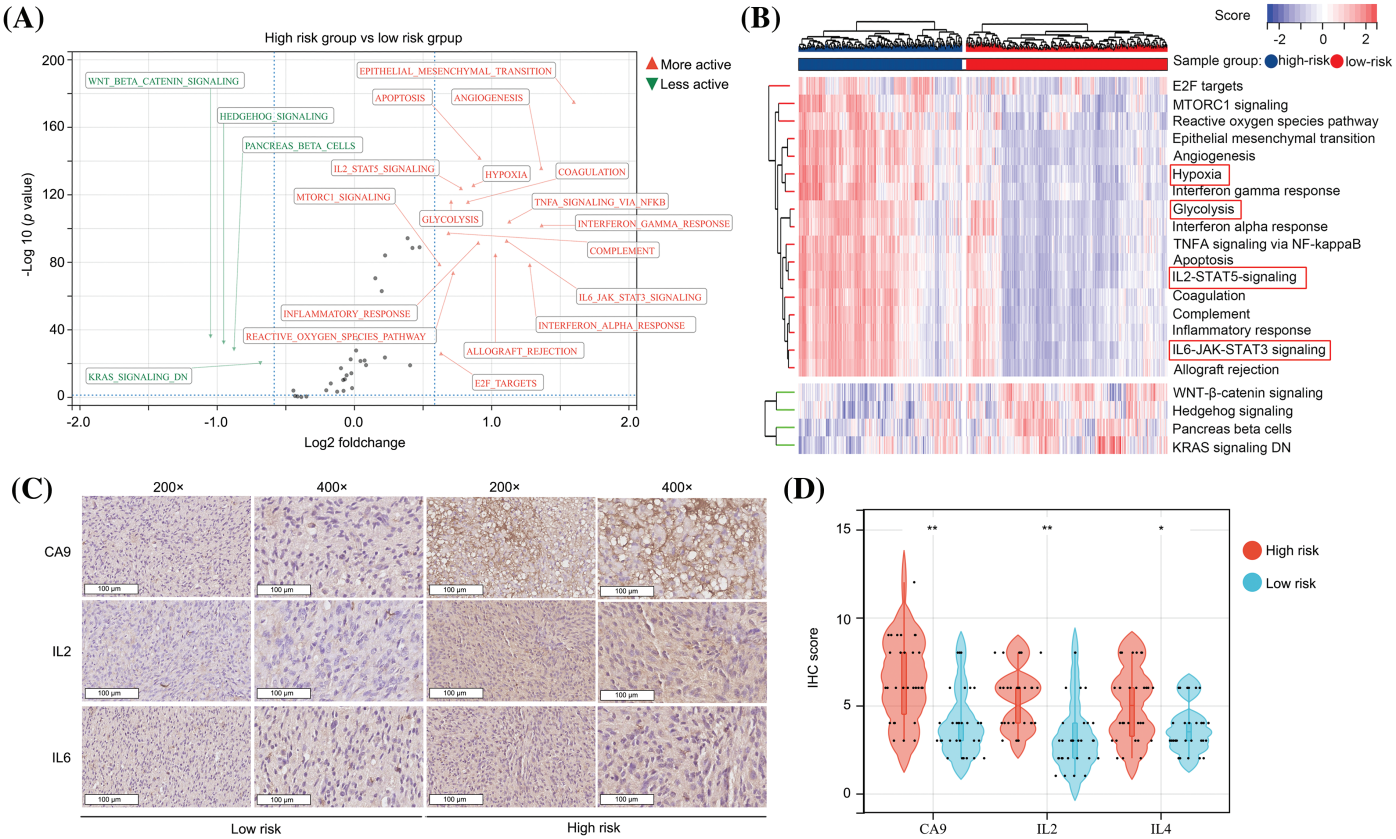
The predictive model has significant diagnostic value for distinguishing high hypoxia and high inflammation microenvironments in glioma tissues. (A and B) GSVA analysis showed the activation and inactivation of pathways in the glioma tissues in the high-risk group. (C and D) IHC was performed in glioma tissues to detect the expression of CA9, IL-2 and IL-6 in the high- and low-risk groups. *p < 0.05; **p < 0.01.

We used GSVA to analyze the molecular signatures of glioma tissues in high- and low-risk groups. It was found that 17 pathways including “E2F targets,” “MTORC1 signaling,” “ROS pathway,” “epithelial-mesenchymal transition,” “angiogenesis,” “hypoxia,” “interferon gamma response,” “glycolysis,” “interferon alpha response,” “TNFA signaling via NF-kappaB,” “apoptosis,” “IL2-STAT5 signaling,” “coagulation,” “complement,” “inflammatory response,” “IL6-JAK-STAT3 signaling” and “allograft rejection” were more activate in the glioma tissues in the high-risk group in comparison to those in the low-risk group (Figs. 5A and 5B), while four pathways, “WNT-β-catenin signaling,” “hedgehog signaling,” “pancreas beta cells” and “KRAS signaling DN” were more inactivate (Figs. 5A and 5B). Most of the pathways that exhibited higher activation in the high-risk group demonstrated cross-talk between inflammation and hypoxia. To validate the findings obtained from the bioinformatic analysis, we conducted IHC assays for CA9 (a biomarker of hypoxia and glycolysis [21]), IL-2, and IL-6 proteins in glioma tissues obtained from our research cohort. The glioma tissues were categorized into high- and low-risk groups based on the risk score calculation (Fig. 4F). The high-risk group displayed elevated expression levels of CA9, IL-2, and IL-6 in the glioma tissues (Figs. 5C and 5D). Taken together, we consider that the predictive model has significant diagnostic value for distinguishing between high hypoxia and high inflammation microenvironments in glioma tissues.

### The predictive model has significant diagnostic value for distinguishing an immunosuppressive TME in glioma tissues

**Figure 6:**
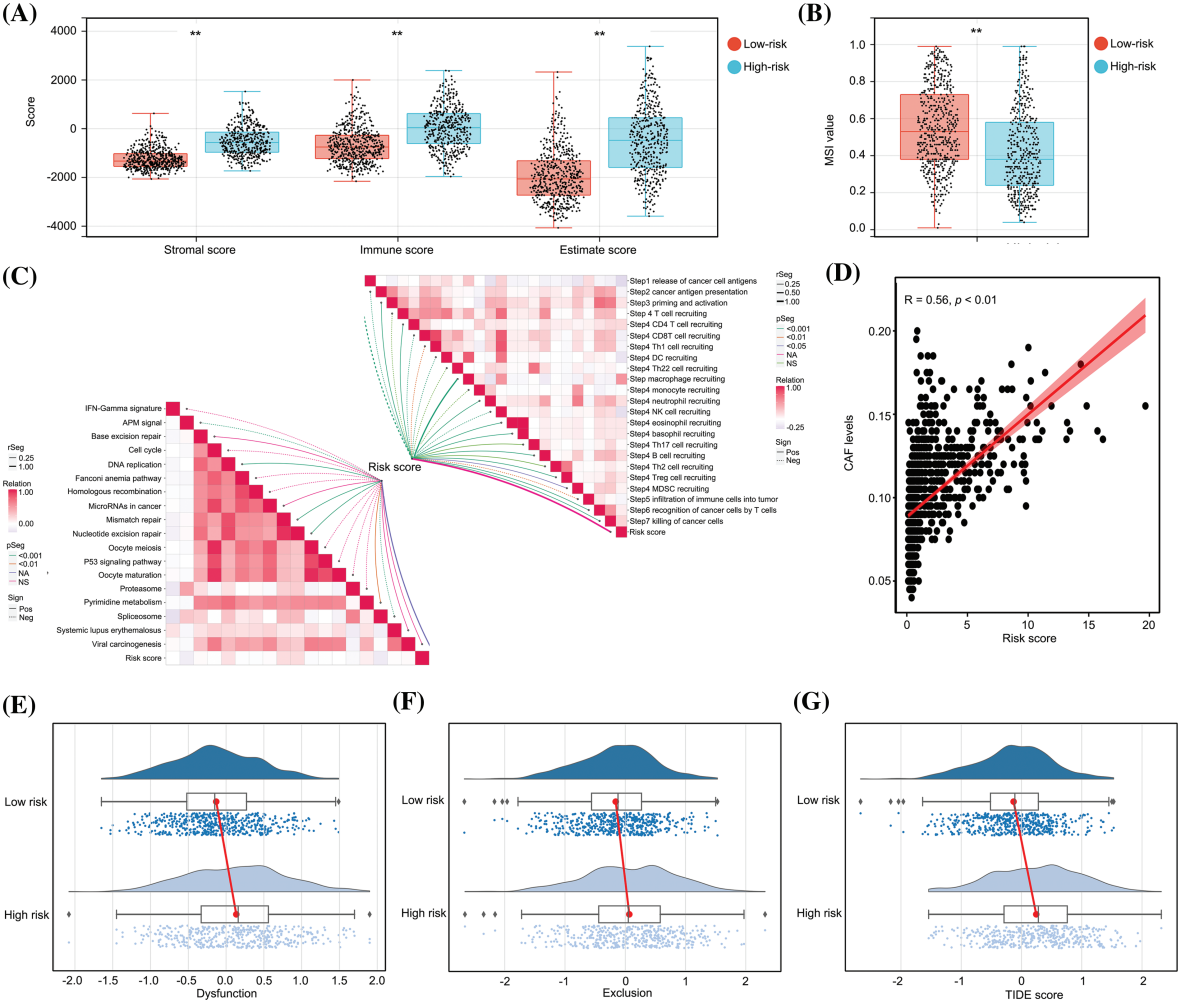
The predictive model had significant diagnostic value for distinguishing an immunosuppressive TME in glioma tissues. (A) Stromal score, immune score and ESTIMATE scores in high- and low-risk group glioma tissues. (B) MSI score in high- and low-risk group glioma tissues. (C) Relationship between risk score and anti-cancer immune processes. (D) Relationship between risk score and levels of CAF cells. (E–G) Dysfunction, exclusion and TIDE scores in high- and low-risk groups. **p < 0.01.

Subsequently, a comparative analysis of the immunological characteristics of low- and high-risk glioma tissues was conducted. An estimation algorithm revealed that glioma tissues from the high-risk group exhibited increased stromal, immune, and ESTIMATE scores (Fig. 6A). Furthermore, glioma tissues obtained from high-risk patients demonstrated a diminished score for microsatellite instability (MSI) in comparison to those obtained from low-risk patients (Fig. 6B). Additionally, we analyzed the relationship between the risk score and immune reaction process according to a previous study [22]. Our study revealed that the risk score was predictive for unsuccessful anti-tumor immune response stages, encompassing the liberation of cancer cell antigens, initiation and activation processes, and recruitment of CD4 and CD8 T cells (Fig. 6C). Furthermore, a positive correlation was observed between the risk score and levels of cancer-associated fibroblasts (Fig. 6D). We also demonstrated that the high-risk group exhibited elevated levels of dysfunction (Fig. 6E), exclusion (Fig. 6F), and TIDE scores (Fig. 6G) within glioma tissues, suggesting a diminished potential for immunotherapy efficacy. In conclusion, the predictive model has substantial diagnostic utility in discerning the immunosuppressive tumor microenvironment in glioma tissues.

### The predictive model can act as indication for optimizing the use of trametinib

**Figure 7:**
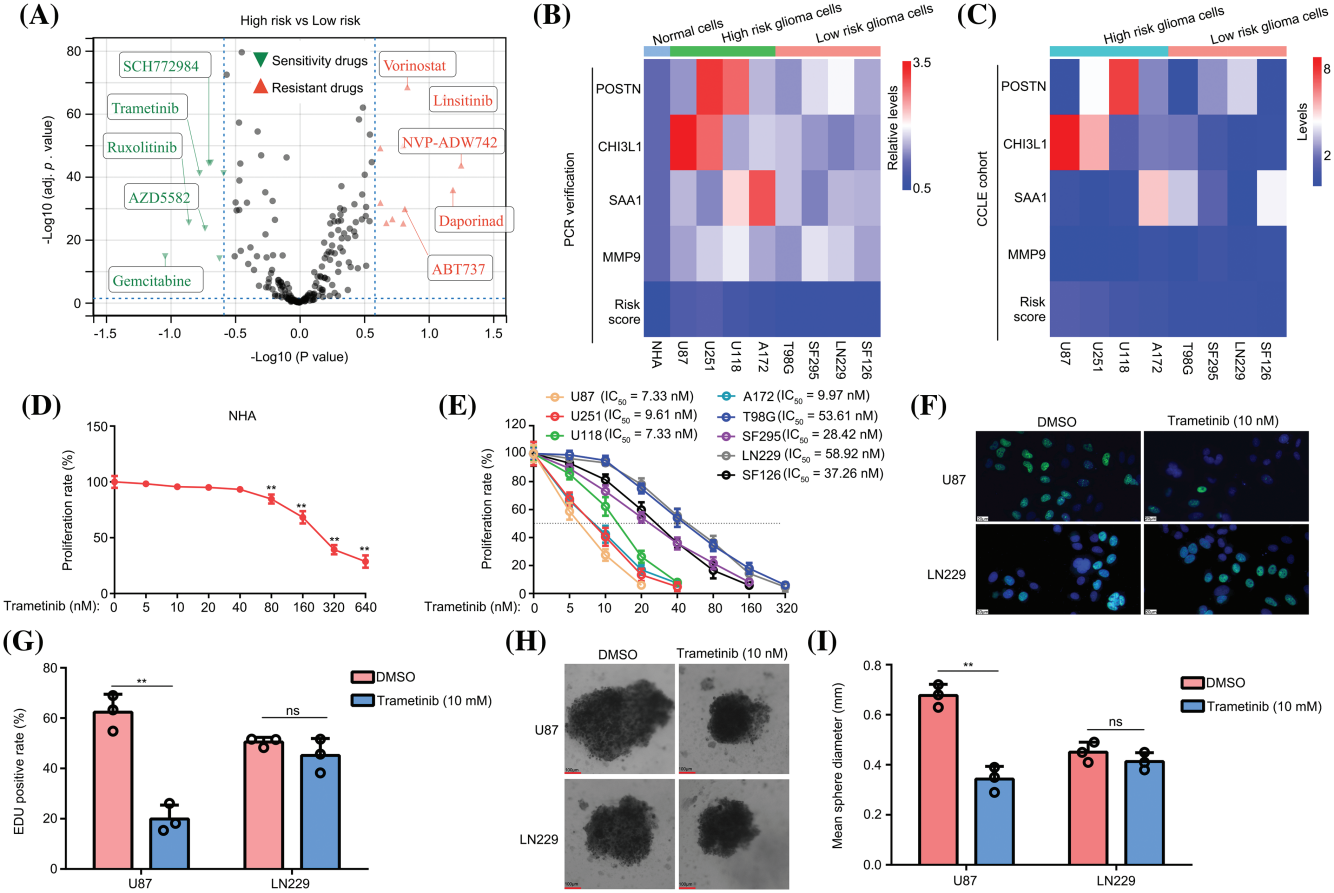
The predictive model can be used to optimize the use of trametinib. (A) OncoPredict was used to determine drug sensitivity and resistance in patients with high- and low-risk gliomas. (B) The expression of *POSTN*, *CHI3L1*, *SAA1*, and *MMP9* was detected in NHA, U87, U251, U118, A172, T98G, SF295, LN229, and SF126 cells using qRT-PCR. (C) Expression of *POSTN*, *CHI3L1*, *SAA1*, and *MMP9* in U87, U251, U118, A172, T98G, SF295, LN229, and SF126 cells in the CCLE data. (D) The nonspecific toxicity of trametinib for NHA was detected by CCK-8 at 48 h. (E) The inhibitory effects of trametinib on U87, U251, U118, A172, T98G, SF295, LN229, and SF126 cells were detected by CCK-8 at 48 h. (F and G) EDU assays were used to detect the inhibitory effects of 10 nM trametinib in U87 and LN229 cells. The white bar mean 20 μm. (H and I) A 3D spheroid model was used to detect the inhibitory effects of 10 nM trametinib on U87 and LN229 cells. **p < 0.01; ns, not significant.

In this study, we employed OncoPredict to examine variations in drug sensitivity between high- and low-risk glioma groups. The findings revealed that the top five drugs exhibiting high-risk score sensitivity in patients with glioma were SCH772984, trametinib, ruxolitinib, AZD5582, and gemcitabine. Conversely, the top five drugs demonstrating low-risk score sensitivity in patients with glioma were vorinostat, linsitinib, NVP-ADW742, daporinad, and ABT737 (Fig. 7A). Unlike other drugs, trametinib has progressed to phase II clinical trials of glioma treatment and has demonstrated significant variations in treatment outcomes among patients [23,24]. Consequently, our objective was to investigate whether this predictive model could serve as an indicator to optimize trametinib utilization. The expression levels of POSTN, CHI3L1, SAA1, and MMP9 were assessed in various glioma cell lines (U87, U251, U118, A172, T98G, SF295, LN229, and SF126) using qRT-PCR. Our findings revealed that all these markers were elevated in glioma cells compared to normal glial cells (NHA; Fig. 7B). After substituting gene expression into the model formula, the cells were artificially divided into two groups based on the calculated risk value: a high-risk group consisting of U87, U251, U118, and A172 cells, and a low-risk group consisting of T98G, SF295, LN229, and SF126 cells (Fig. 7B). To ensure accuracy, we compared the gene expressions of *POSTN*, *CHI3L1*, *SAA1*, and *MMP9* in these glioma cells using the CCLE database and confirmed that the classification was consistent (Fig. 7C).

Subsequently, we used the CCK-8 method to assess the nonspecific toxicity and inhibitory ability of trametinib in NHA and glioma cells. The findings of this study revealed that trametinib exhibited no nonspecific cytotoxicity towards normal astrocytes (NHA) within a concentration range of 40 nM (Fig. 7D). Moreover, the IC50 values of trametinib were below 10 nM in glioma cells belonging to the high-risk group (U87, U251, U118, and A172), whereas the IC50 values ranged from to 30–70 nM in glioma cells from the low-risk group (T98G, SF295, LN229, and SF126) (Fig. 7E). These findings indicate that glioma cells in the low-risk group exhibited a lack of sensitivity to trametinib. The administration of a high dose of trametinib demonstrated a notable inhibitory effect on the low-risk group cells but also induced significant toxicity in NHA cells.

Subsequently, U87 cells from the high-risk group and LN229 cells from the low-risk group were selected for further analysis. Using the EDU assay, we observed that a concentration of 10 nM trametinib effectively reduced the EDU positive rate in U87 cells, whereas it did not have the same effect in LN229 cells (Figs. 7F and 7G). Moreover, using a 3D spheroid model, we demonstrated that 10 nM trametinib effectively suppressed the proliferation of U87 cells under 3D conditions. However, this inhibitory effect was not observed in LN229 cells (Figs. 7H and 7I). Collectively, these results suggest that the predictive model can be used to optimize the use of trametinib.

## Discussion

Glioma, a prevalent primary brain tumor in adults, is associated with a poor prognosis. Despite the implementation of various treatments, such as immunotherapies, chemotherapies, and targeted therapies, their efficacy is hindered by the heterogeneous TME characteristics of glioma [25,26]. Consequently, understanding and controlling the intricate interplay between the diverse constituents of the TME may help in the diagnosis and treatment of gliomas.

Immune cells play a crucial role in the glioma TME, as they can be selectively recruited or activated by various factors present in the TME, including hypoxia, acidosis, and inflammatory factors. Subsequently, these immune cells engage in cross-talk with tumor cells, thereby influencing a range of malignant behaviors exhibited by tumor cells such as proliferation, invasion, and drug resistance [27,28]. Furthermore, immune cells possess the ability to respond to the microenvironment, potentially exacerbating hypoxia and inflammation [13,14]. Based on the aforementioned information, our initial focus was directed towards immune cells and immune cell-related genes within the glioma microenvironment, with the aim of identifying genes that could serve as markers for glioma. In the present study, we observed that M2 macrophages exhibited the highest abundance among immune cells within the microenvironment of glioma tissues, and this abundance was positively correlated with poor prognosis. Additionally, we identified eight genes, namely *IL-8*, *POSTN*, *CHI3L1*, *SAA1*, *PLA2G2A*, TREM1, *IBSP*, and *MMP9*, that were upregulated in glioma tissues with high levels of M2 macrophages.

Cellular communication is a multifaceted and intricate process wherein cross-talk can be facilitated through diverse mechanisms such as mRNA transfer, ligand-receptor interactions, and the release of secretory factors [29,30]. Single-cell sequencing is a valuable approach to investigate the genomic and transcriptomic profiles of individual cells. When coupled with a range of analytical techniques, it enables the elucidation of intricate modes of communication between cell populations within tumor tissues, as well as the identification of key mediators of this communication [31]. To gain deeper insight into the functions of the aforementioned eight genes, we performed scRNA sequencing. Our findings indicate that these genes are predominantly expressed in both TAM and tumor cells, suggesting their potential significance in facilitating intercellular communication between tumor cells and TAM.

The genes *POSTN*, *CHI3L1*, *SAA1*, and *MMP9* were screened to establish a predictive model, which exhibited a significant correlation with glioma prognosis and served as independent prognostic factors. Previous studies have constructed prediction models based on other signatures in gliomas, such as ferroptosis- and pyroptosis-related genes [32,33]. However, the prognostic value (AUC) of most prediction models ranged from 0.65 to 0.75. In contrast, the prognostic value of the prediction models constructed in our study exceeded 0.80 for prognosis. This may be one of the strengths of our model, and we believe that the model can serve as a significant biomarker to predict the prognosis of gliomas.

Multiple genetic alterations in glioma tissues, such as *IDH1* mutations, 1p/19q co-deletion, and *MGMT* promoter methylation, serve as significant indicators of prognosis and drug responsiveness [19]. In clinical practice, the combination of the 1p/19q co-deletion and *MGMT* promoter methylation is considered the “gold standard” for assessing the sensitivity of gliomas to TMZ. Gliomas lacking both the 1p/19q co-deletion and *MGMT* methylation commonly exhibit TMZ resistance [20]. Therefore, we present the second advantage of our predictive model, which can reflect the mutational status of gliomas, wherein high-risk patients exhibit a lower frequency of *IDH1* mutation, 1p/19q co-deletion and *MGMT* methylation.

Moreover, our study revealed that the expression levels of four genes (*POSTN*, *CHI3L1*, *SAA1*, and *MMP9*) were significantly elevated in glioma samples obtained from patients who experienced recurrence within one year after TMZ treatment, along with an increased risk score. Previous studies have extensively documented the dysregulation and oncogenic properties of *POSTN*, *CHI3L1*, *SAA1*, and *MMP9* in various cancer types, including gliomas. *POSTN* encodes an extracellular matrix protein that plays a crucial role in tissue regeneration and development [34]. Additionally, the protein encoded by *POSTN* contributes to the maintenance of stemness and promotes metastasis [35]. The *POSTN*-encoded protein in gliomas serves as an inducer of M2 macrophage recruitment, thereby inducing an immune-suppressive TME and facilitating the proliferation of glioma cells [36]. The *CHI3L1*-encoded protein, belonging to the glycoprotein family, is secreted by macrophages during inflammation an tissue remodeling [37]. Steponaitis et al. conducted a study revealing elevated mRNA levels of CHI3L1 in glioma tissues compared to adjacent tissues, and this increased CHI3L1 expression was found to be associated with poor prognosis in patients with glioma [38]. A previous study indicated that cancer cells secrete CHI3L1 into the TME and recruit M2 macrophages for reprogramming [39]. *SAA1* encodes an apolipoprotein within the serum amyloid A family that is highly expressed in response to inflammation and tissue damage [40]. Zhang et al. showed that the suppression of SAA1 can effectively reduce the mobility of glioma cells and enhance their sensitivity to TMZ [41]. *MMP9* encodes a secreted protein that plays a role in the degradation of the extracellular matrix [42]. Previous studies have indicated that MMP9 can be secreted by M2 macrophages within the glioma tissue microenvironment, thereby promoting cellular invasion [43]. In summary, previous research has established the significance of these four genes as biomarkers of gliomas.

Previous research has demonstrated that the induction of an immunosuppressive environment in glioma tissues is facilitated by both hypoxia and activation of inflammation-related pathways. This is achieved by the recruitment of circulating macrophages and their subsequent polarization to the M2 phenotype [44,45]. These findings suggest that cross-talk between glioma cells and macrophages can provide insights into the characteristics of the microenvironment. In accordance with this, our study revealed that the predictive model we developed, based on the interaction of cross-talk genes between TAM and glioma cells, exhibited the capability to discern the TME characterized by elevated hypoxia, inflammation, and immunosuppression.

In our analysis of drug sensitivity in patients with glioma or cells categorized as high- or low-risk, we identified that the high-risk group showed sensitivity to trametinib. Trametinib, an orally effective mitogen-activated protein kinase (MEK) inhibitor, inhibits various cancer cells by reducing phosphorylated MEK levels [46,47]. Furthermore, trametinib has been observed to effectively penetrate the blood-brain barrier and exert an anti-tumor effect in the target area, as evidenced by several phase II clinical studies conducted on gliomas. However, the efficacy of trametinib in patients varies with outcomes ranging from complete response to disease progression [48,49]. Unfortunately, there is currently a lack of established indications for the appropriate use of trametinib. The predictive model developed in this study may serve as a tool for optimizing the selection of suitable candidates for trametinib treatment.

## Conclusion

In conclusion, our study demonstrated a noteworthy correlation between M2 macrophages and an unfavorable prognosis in glioma, highlighting the significant impact of cross-talk between macrophages and cancer cells on the composition of the microenvironment in glioma tissues. The predictive model constructed using four genes involved in macrophages and cancer cells, namely *POSTN*, *CHI3L1*, *SAA1*, and *MMP9*, holds promise in distinguishing high inflammation-hypoxia-immunosuppression microenvironment signatures and helps optimize trametinib selection for glioma treatment.

Our findings align with existing knowledge, as we observed a strong association between these biomarkers and TMZ resistance, as well as their substantial diagnostic potential in predicting recurrence following TMZ treatment. Moreover, our study demonstrated that the predictive model incorporating all four biomarkers exhibited superior diagnostic efficacy compared with individual biomarkers. This may be another advantage of our prediction model, indicating that it can act as a biomarker to optimize the application of TMZ in patients with gliomas. However, our study has certain limitations that must be acknowledged. First, although all patients in our validation set underwent radiation therapy, the sample size was insufficient to ascertain the effect of varying radiotherapy doses and sites on the outcomes. Furthermore, our investigation focused solely on molecular phenotypes without incorporating models that integrate clinical staging with molecular phenotypes. Hence, in the subsequent phase, we intend to implement two strategies: firstly, incorporating a larger cohort of patients who have undergone conventional TMZ treatment and categorizing them into distinct radiation dose groups to investigate the potential utility of the identified genes as indicators for relapse in varying radiation dose groups; secondly, enrolling a more diverse population of glioma patients at different stages and evaluating the diagnostic efficacy of our prognostic model for each stage, while ensuring the comparability of baseline clinical characteristics.

## Supplementary Materials







## Data Availability

Corresponding authors can be contacted to obtain data when necessary.

## Supplementary Materials

The supplementary material is available online at https://doi.org/10.32604/or.2024.046191.
